# Mathematical modeling of dopamine rhythms and timing of dopamine reuptake inhibitors

**DOI:** 10.1371/journal.pcbi.1013508

**Published:** 2025-09-25

**Authors:** Tianyong Yao, Ruby Kim

**Affiliations:** 1 Department of Mathematics, University of Michigan, Ann Arbor, Michigan, United States of America; 2 Department of Anesthesiology, Michigan Medicine, Ann Arbor, Michigan, United States of America; University of Bath, UNITED KINGDOM OF GREAT BRITAIN AND NORTHERN IRELAND

## Abstract

Dopamine (DA) plays a vital role in mood, alertness, and behavior, with dysregulation linked to disorders such as Parkinson’s disease, ADHD, depression, and addiction. In this study, we develop and analyze a reduced mathematical model of dopamine synthesis, release, and reuptake to investigate how daily rhythms influence dopamine dynamics and the efficacy of dopamine reuptake inhibitors (DRIs) used in the treatment of various neuropsychiatric conditions. We simplify a detailed mathematical model of dopamine synthesis, release, and reuptake and demonstrate that our reduced system maintains key dynamical features including homeostatic regulation via autoreceptors. Our model captures core autoregulatory mechanisms and reveals that DRIs can exert substantial time-of-day effects, allowing for dopamine levels to be sustained at elevated levels when administered at circadian troughs. These fluctuations depend sensitively on the timing of DRI administration relative to circadian variations in enzyme activity. We further extend the model to incorporate feedback from local dopaminergic tone, which generates ultradian oscillations in the model independent of circadian regulation. Administration of DRIs lengthens the ultradian periodicity. Our findings provide strong evidence that intrinsic fluctuations in DA should be considered in the clinical use of DRIs, offering a mechanistic framework for improving chronotherapeutic strategies targeting dopaminergic dysfunction.

## Author summary

Dopamine levels can fluctuate throughout the day, and these changes are in part regulated by the circadian clock. Many drugs prescribed for neurological or psychiatric conditions influence dopamine metabolism, but their time-of-day effects are not well understood. Using mathematical modeling, we present evidence of time-of-day effects of dopamine reuptake inhibitors like modafinil or bupropion. We find in the model that taking doses at the wrong time of day can cause large spikes and subsequent crashes in dopamine levels, while strategic timing can sustain dopamine levels for much longer. Our model can be used to explore the outcomes of different dose schedules and to inform treatment strategies. In addition, we find that incorporating population-level activity of dopaminergic neurons generates intrinsic, 4-hour ultradian rhythms that are separate from circadian input. Dopamine fluctuates periodically even without a circadian drive, but the underlying mechanisms have not been understood. Our model provides a plausible explanation of dopamine ultradian oscillations as a neuronal population-level phenomenon.

## 1. Introduction

Dopamine (DA) levels in the brain fluctuate throughout the day, influencing alertness, mood, and decision-making. DA dysfunction is associated with numerous health issues. A primary characteristic of Parkinson’s disease (PD) is the loss of dopaminergic neurons in the substantia nigra [1–3]. Various drugs of abuse stimulate dopamine release and create drug dependence [4]. Dopamine dysregulation has been linked to depression, schizophrenia, and attention deficit hyperactivity disorder (ADHD) [5,6]. These health consequences highlight the importance of dopamine regulation in the brain.

Intracellular and extracellular concentrations of dopamine are tightly modulated by autoregulatory feedback mechanisms. In a dopaminergic cell, the enzyme tyrosine hydroxylase (TH) converts tyrosine into levodopa, which is then decarboxylated into DA. This intracellular DA is then packaged into vesicles and released into the extracellular space. Dopamine D2 autoreceptors encoded by the *DRD2* gene inhibit tyrosine hydroxylase activity to regulate synthesis, and the dopamine transporter (DAT) moves excess extracellular DA back into the neuron [7,8]. Mathematical modeling shows that DA homeostasis relies on complex interactions between these mechanisms [9–11].

A better understanding of these biochemical pathways may lead to critical health insights. *DRD2* is significantly associated with sleep disruptions [12] and has been identified as the gene most strongly linked to self-reported tiredness in a genome-wide association study [13]. Gene-set association analyses have identified significant associations between DA-related genes and ADHD, autism spectrum disorder, bipolar disorder, major depression, Tourette’s syndrome, and schizophrenia [14]. Dysregulation of DAT has been implicated in Parkinson’s disease, ADHD, bipolar disorder, and depression [15]. In addition, drugs acting on dopaminergic pathways are commonly prescribed to alleviate symptoms of various health conditions. For example, modafinil inhibits DAT activity, which increases extracellular concentrations of dopamine and promotes wakefulness in individuals experiencing fatigue or excessive daytime sleepiness [16]. Methylphenidate prescribed for ADHD and bupropion used to treat depression, addiction, and seasonal affective disorder (SAD) also act as dopamine reuptake inhibitors (DRIs), a class of drugs that inhibit DAT activity [17].

While external stimuli like reward and stress can cause transient changes, endogenous circadian rhythms drive roughly 24-hour periodic behavior in dopamine synthesis, reuptake, and release in individual dopaminergic neurons. In general, dopamine plays an important role in sleep, wakefulness, and other rhythmic processes [18,19]. For example, activation of dopaminergic neurons in the ventral tegmental area (VTA) promotes wakefulness [20], and dopamine signaling in the suprachiasmatic nucleus (SCN), the central circadian pacemaker, can phase-shift activity patterns in mice [21,22]. Previous mathematical models [23,24] have been used to investigate the molecular mechanisms that produce circadian rhythms in the dopaminergic system in mammals [25–28], and to study reciprocal phase-shifting effects [29].

**Fig 1:**
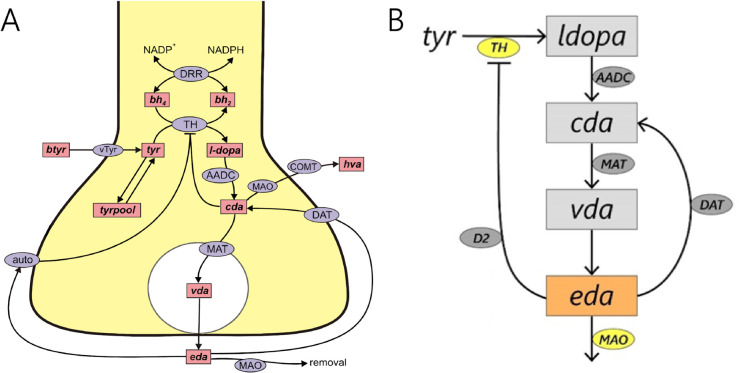
Schematic diagram of full and reduced mathematical models. (A) Schematic diagram of full model of dopamine synthesis, release, and reuptake by Best et al. [9]. Figure modified from [9]. Abbreviations: btyr, blood tyrosine; bh2, dihydrobiopterin; bh4, tetrahydrobiopterin; tyr, tyrosine; l-dopa, l-3,4-dihydroxyphenylalanine; cda, cytosolic dopamine; vda, vesicular dopamine; eda, extracellular dopamine; hva, homovanillic acid; trypool, the tyrosine pool; vTyr, neutral amino acid transporter; DRR, dihydrobiopterin reductase; TH, tyrosine hydroxylase; AADC, aromatic amino acid decarboxylase; MAT, vesicular monoamine transporter; DAT, dopamine transporter; auto, D2 dopamine auto receptors; MAO monoamine oxidase; COMT, catecholamine O-methyl transferase; NADP, nicotinamide adenine dinucleotide phosphate; NADPH, nicotinamide adenine dinucleotide phosphate, reduced form. (B) Tyrosine (*tyr*) is converted into levodopa (*ldopa*), which is decarboxylated to make cytosolic dopamine (*cda*). Cytosolic dopamine is then packaged into vesicles (vesicular dopamine, *vda*) and released into the extracellular space as extracellular dopamine (*eda*). The state variables of the model equations are in rectangles, and enzymes that determine reaction rates are in ellipses. Enzymes that are influenced by the molecular clock in the model are highlighted in yellow.

Many drugs taken for neuropsychiatric or neurodegenerative disease, including DRIs, target proteins or functions known to be regulated by the molecular clock, but circadian timing is not often considered in treatment protocols [30]. There is strong evidence of chronotherapeutic effects of antidepressants in both rodents and humans, with greater efficacy at certain times of the day depending on the drug [31–34]. However, in general, very few experimental or clinical studies have investigated the effects of time of day on drugs targeting neurotransmission. In this paper, we reduce an existing mathematical model of dopamine synthesis, release, and reuptake [9] to four core variables, allowing us to analytically compute equilibria and determine their asymptotic stability; see Fig 1 for a model schematic. We demonstrate that the reduced model retains essential dynamics of the full model and use the model to explore time-of-day effects of DRIs targeting the dopaminergic system. We found that the administration time had a substantial impact on the time course of DA, with sustained elevation of DA levels when administered at circadian troughs and large fluctuations when administered at circadian peaks.

In addition, the dopaminergic system is known to display ultradian rhythms with periods ranging from 1-6 hours [35,36]. Ultradian rhythms are fundamental to various physiological processes, including hormone secretion, sleep stages, and behavioral arousal [37–39]. In mammals, ultradian rhythms play a crucial role in organizing behavioral activity and enhancing responsiveness to environmental stimuli, thereby contributing to survival and adaptive behaviors. Inhibiting dopamine reuptake lengthens the period of these ultradian rhythms [39]. Previous studies hypothesized that DA self-feedback via D2 autoreceptors can generate ultradian oscillations [40]. We extend our reduced model to include sensing of overall extracellular DA produced by a local population of neurons. This coupling introduces intrinsic delays in autoregulatory mechanisms, enabling the emergence of ultradian dopamine rhythms. Within this framework, we also examine how DRIs might influence the period of ultradian rhythms.

In Sect 2.1, we discuss how circadian rhythms influence the dynamics of the dopaminergic system. In Sect 2.2, we study the effects of drug administration time of DRIs on the dynamical behaviors of intracellular and extracellular dopamine in the model, explaining why certain administration times cause larger daily fluctuations in dopamine. In Sect 2.3 we investigate the stability of the model solutions for a wide range of drug half-lives and inhibitory effects. Finally, in Sect 2.4, we develop a Dopamine Ultradian Oscillator (DUO) model by introducing an *eda* pool that accumulates dopaminergic output from neuron terminals and feeds back via D2 autoreceptors, enabling intrinsic ultradian dopamine rhythms. In Sect 2.5, we investigate how DRIs and other parameters in the DUO model modulate the resulting oscillatory periodicity and amplitude in the absence of circadian input. The MATLAB code available at https://github.com/rubyshkim/YaoKim_DA can be used for *in silico* experiments to form new hypotheses about chronotherapeutic effects of drugs acting on the dopaminergic system.

## 2. Results

We reduced the mathematical model in [9] from 9 differential equations to only 4 by focusing on the dynamics between levodopa (*ldopa*), cytosolic dopamine (*cda*), vesicular dopamine (*vda*), and extracellular dopamine (*eda*). The reduction allows for detailed analyses of the dynamical behaviors as well as large-scale computations, including parameter sweeps. In the dopaminergic terminal, *ldopa* is formed from tyrosine via the tyrosine hydroxylase (*TH*) enzyme-catalyzed reaction. Then, it is decarboxylated to form *cda*, which is then packaged into vesicles as *vda* and released into the extracellular space as *eda*. At high concentrations, *eda* feeds back to inhibit *TH* activity. For large enough *DAT* activity, *eda* is remarkably robust to changes in *TH* activity; see Sect 4.3 in the Methods. We see that there is a homeostatic region where changes in *TH* or *DAT* activity do not significantly impact *eda*, but outside of this region *eda* can become highly sensitive to the activity of these enzymes. We describe the model reduction and compare it to the full model in more detail in Sect 4.3.

Having confirmed that our reduced model reproduces the behavior in [9] well, the sensitivity to *DAT* activity outside of the homeostatic region motivated us to study the effects of DRIs that target and inhibit *DAT* and to investigate time-of-day effects in Sects 2.1, 2.2, and 2.3. In Sect 2.3, we explore a wide range of dosing times and doses. In Sects 2.4, 2.5 we extend the reduced model to incorporate feedback from a local dopamine pool and find that these mechanisms are enough to generate flexible ultradian rhythms.

### 2.1. Circadian rhythms of dopamine synthesis

**Fig 2:**
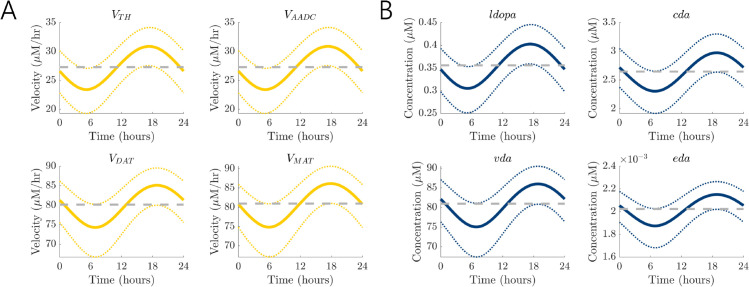
Circadian rhythms of dopamine synthesis, release, and reuptake. The variables and reaction rates in the model display circadian rhythms due to the influence of the molecular clock on *TH* and *MAO* activity. (A) Circadian rhythms of reaction rates (*μ*M/hr). (B) Circadian rhythms of variable concentrations (*μ*M). For both panels, solid curves correspond to time-dependent circadian variation 25% below and above nominal $V_{TH}$ and $V_{MAO}$. Dotted curves correspond to circadian variation relative to 0.75 and 1.25 of nominal $V_{TH}$ and $V_{MAO}$, demonstrating robustness of the model behavior to shifts in the baseline of variation. Dashed gray lines indicate nominal steady state values.

We have previously created mathematical models of circadian rhythms in dopamine produced by regulation of tyrosine hydroxylase (*TH*) and monoamine oxidase (*MAO*), involved in synthesis and degradation respectively, by the molecular clock [23,24], having further extended the models to study reciprocal influences of dopamine and melatonin on circadian rhythms [29,41]. Animal studies have revealed circadian rhythms in *TH* levels across different brain regions [27,42,43] with REV-ERB circadian nuclear receptors repressing *TH* gene transcription [27]. The *MAO* enzyme also varies with circadian rhythms, with gene transcription activated by the Brain and muscle arnt-like (BMAL1) clock protein [25]. In the midbrain, the clock proteins BMAL1 and REV-ERB are antiphasic [27] and in our mathematical models [23,24] we found that these mechanisms generate a nearly antiphasic relationship between circadian rhythms of *TH* and *MAO* activity. As a result, in this study we assumed time-dependent circadian variation in *TH* activity and *MAO*-mediated catabolism, $V_{TH}$ and $V_{CATAB}$, to study circadian time-dependent administration of drugs that target the dopaminergic system. We varied the enzyme activities sinusoidally, multiplying $V_{TH}$ by $C_{TH}(t)=0.25\sin\left(\frac{\pi }{12}(t-12)\right)+1$ and $V_{CATAB}$ by $C_{MAO}(t)=0.25\sin\left(\frac{\pi }{12}(t-20)\right)+1$. The phases were chosen so that the phase gap is 8 hours as predicted by our previous model [23]. Both curves were simplistically chosen to vary from 0.75 to 1.25, so that the activity varies 25% below and above. This amplitude is close to those measured in our detailed circadian-dopamine model with curves fit to animal data, where the amplitude of *TH* mRNA levels was about 0.5 of its peak value and the amplitude of *MAO* activity was about 0.2 of its peak value [23]. The phase shifts were chosen so that $V_{TH}$ reaches its maximum 18 hours into a 24-hour cycle and $V_{CATAB}$ peaks roughly 8 hours later, as observed in earlier simulations [23]. In the next section where we study dose timing, the timing will be relative to these choices in phase shift. We note, however, that *C*_*MAO*_ had relatively little effect on the model solutions, and periodic behaviors are effectively driven by *C*_*TH*_. With time-dependent changes in *TH* and *MAO* activity, there are corresponding circadian rhythms in reaction velocities and variable concentrations; see Fig 2. The rate of conversion of *ldopa* to *cda* is $V_{AADC}\approx$ 30 *μ*M/hr while $V_{MAT}\approx 80$ *μ*M/hr. As a result, *cda* is packaged into vesicles much more quickly than it is synthesized. In addition, the nominal model has a constant release rate of fire(t)=1, so *vda* is released as *eda* at a rate of about 80 *μ*M/hr. Because the packaging and release steps occur at similar rates in the model (≈80 *μ*M/hr), the time profiles of *vda* and *eda* closely follow that of *cda*. Thus, while the peak in *ldopa* occurs 17.169 hours into the cycle, *cda*, *vda*, and *eda* all peak 1.55 hours later. In addition, it is known that enzyme expression varies by about 25% between individuals of the same species [44–46], and we find that the qualitative behavior is robust to 25% shifts in the baseline of circadian variation; see the dotted curves in Fig 2.

### 2.2. Critical timing of dopamine reuptake inhibitors

**Fig 3:**
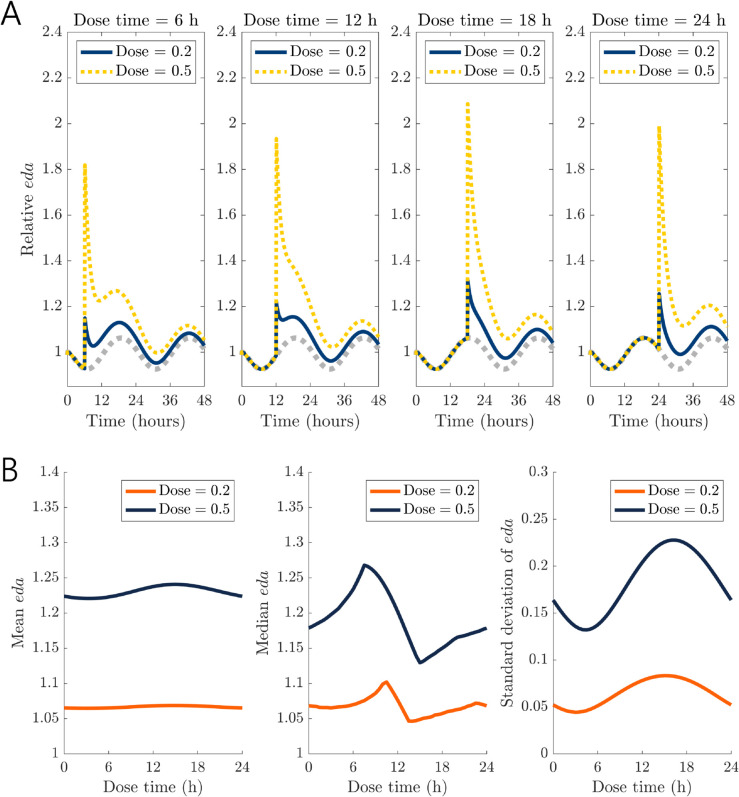
Single-dose administration of a dopamine reuptake inhibitor (DRI) at different times of the day. (A) The plotted doses initially block either 20% or 50% of the dopamine transporters and decay exponentially with a half-life of 15 hours. The time of administration (6, 12, 18, or 24 hours into the day) has a substantial influence on the *eda* curve, which is plotted relative to its nominal steady-state concentration in the absence of drug. A gray dotted line shows the time course of *eda* in the absence of drug and serves as a reference trajectory. *Dose* = 0.2 or 0.5 at a single administration time *t* = 6, 12, 18, or 24, going left to right. (B) Time-dependent efficacy of DRIs. During the 24 hours following a single dose of a DRI, the effects on mean *eda* are minimal. However, there are substantial effects of dose time on the median and standard deviation of *eda*. Solid curves correspond to an initial DAT occupancy of 20% and dashed curves correspond to 50% occupancy and the DRI half-life is taken to be 15 hours as in the previous panel. *Dose* = 0.2 or 0.5 at single administration times throughout the entire day.

In this section, we demonstrate that circadian rhythms in the enzyme activity in the model create time-dependent changes in the efficacy of reuptake inhibitors. A class of drugs called dopamine reuptake inhibitors (DRIs) increases extracellular dopamine concentrations by binding to dopamine transporters (DATs) and inhibiting the reuptake of dopamine into the cell. As detailed in the Methods, we modeled the effect of DRIs as a fraction 1−*x*_*dose*_(*t*) multiplied to the velocity of the DAT reaction, $V_{DAT}$. In the model, *x*_*dose*_ increases instantaneously at some administration time and decays exponentially. When *x*_*dose*_ = 0, there is no effect in the model, and when $0<x_{dose}\leq 1$, the velocity of the reaction is reduced. We tested various experimental conditions, summarized in Table 3 in the Methods. To test for circadian variation in DRI efficacy, we introduced doses of 0.2 and 0.5 (reducing $V_{DAT}$ by 20% and 50%) administered at *t* =  6, 12, 18, and 24; see Fig 3. We assumed a half-life of 15 hours, close to the half-life of modafinil which is used to promote wakefulness [47]. Throughout this section, we assume modafinil-like kinetics, and in Sect 2.3 we experiment with half-lives. The administration of the DRI causes a large spike in *eda*, which falls back down over the course of the day.

We find that the administration time of DRIs has a substantial effect on the dynamical behavior of dopamine in the model. The inhibition of dopamine reuptake causes *eda* to rapidly increase and *ldopa*, *cda*, and *vda* to decrease. When the DRI is administered at a time when all model variables are elevated (e.g. *t* = 18), even though extracellular dopamine levels go up rapidly, the rate of the *TH* reaction, $V_{TH}$, is on its way back down due to circadian rhythms. Because the production of intracellular dopamine is decreasing over the next few hours, *eda* levels are not sustained.

To summarize the time-dependent effects of DRIs, we computed the mean, median, and standard deviation of *eda* during the 24-hour period following each dose. These metrics capture different aspects of the system’s response: the mean reflects the overall magnitude of elevation, the median provides a measure of the typical *eda* level over the 24-hour window, and the standard deviation indicates variability in the response profile. In particular, a higher median suggests that *eda* remains elevated for a substantial portion of the 24-hour period. Together, these metrics offer a compact and interpretable summary of how DRI efficacy depends on the time of administration.

We found that the largest changes in mean or median *eda* over the next 24 hours do not necessarily come from large spikes in the concentration. We tested dose times every half-hour from *t* = 0 to *t* = 24 and found that the mean *eda* during the 24-hour period following administration did not change significantly; see Fig 3B. Interestingly, the median *eda* varied significantly from 12.9% to 26.8% above steady state for *Dose* = 0.5. The standard deviation of *eda* over the 24-hour period following administration was also sensitive to dose time. When DRIs are administered when *eda* is elevated and before dopamine synthesis naturally decreases due to circadian rhythms, the boost in *eda* is large but short-lived (e.g. <6 hours). When DRIs are administered before the natural increase in dopamine synthesis, the *eda* boost is relatively small but the circadian drive kicks in and *eda* stays elevated longer. Dopamine is essential for executive functions [48], and the time-of-day effects predicted by our model are consistent with clinical studies measuring cognitive performance. Modafinil administered during circadian troughs (early morning) had the most noticeable effect on alertness and performance on cognitive tasks [49,50], with efficacy lasting about 10 hours despite its half-life of 10-15 hours [49].

**Fig 4:**
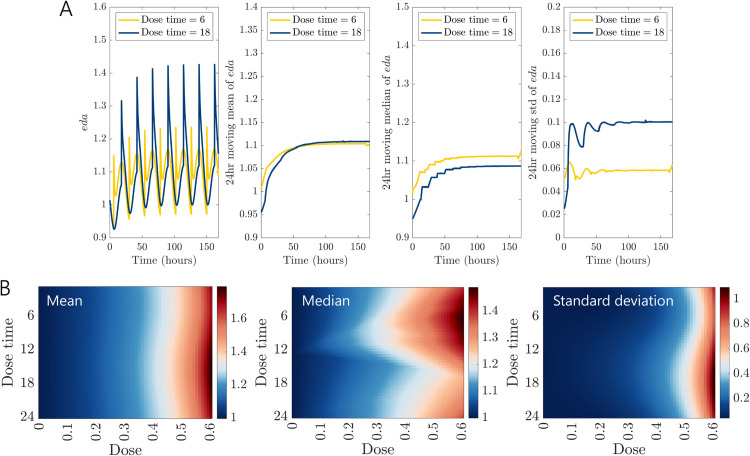
Repeated doses of DRIs at different times of the day. (A) A DRI dose is given at the same time every day, 6 hours into the day (yellow curve) or 18 hours into the day (blue curve) for 7 days. Both dosing schedules elevate the 24-hour moving mean and median of *eda* relative to the nominal steady state concentration over the course of several days. Though repeated doses at 18 hours cause initial spikes in *eda*, the 24-hour moving median remains consistently lower over the following 7 days compared to dosing at 6 hours, indicating that *eda* stays elevated for a larger portion of each 24-hour period when doses are given at 6 hours. The 24-hour standard deviations indicate that *eda* is much more variable throughout a 24-hour period with the later administration time. *Dose* = 0.2 for administration times *t*_*i*_ = 6 + 24(*i*−1) (yellow curve) or *t*_*i*_ = 18 + 24(*i*−1) (blue curve) for i=1,2,...,7. (B) Heat maps of mean, median, and standard deviation across 7 days of repeated doses for varying doses and dose times.

With repeated doses of *Dose* = 0.2 at the same time each day, we find that the time of administration has a substantial influence on the dynamical behavior of dopamine in the long run as well. Repeated daily doses increase the concentration of *eda* on average over time; see Fig 4A. As in our simulations with a single dose, administration at *t* = 18 quickly increased *eda* substantially (by more than 20%) after the first dose. With repeated doses, the spike in *eda* went up to 40% above the steady state value. We found that the time of administration did not have a significant influence on the long-term change in the moving mean over time, but that it influenced the shape of the *eda* curve like in our single dose simulations. The 24-hour moving median and moving standard deviation were both sensitive to dose time, a pattern we found across a wide range of doses; see Fig 4B. While we might not see noticeable changes in the concentration of *eda* on average, the dynamical behaviors are notably different across dose administration times, with larger daily fluctuations in *eda* for dose times between *t* = 12 and *t* = 24.

### 2.3. Stable dosing regimes

**Fig 5:**
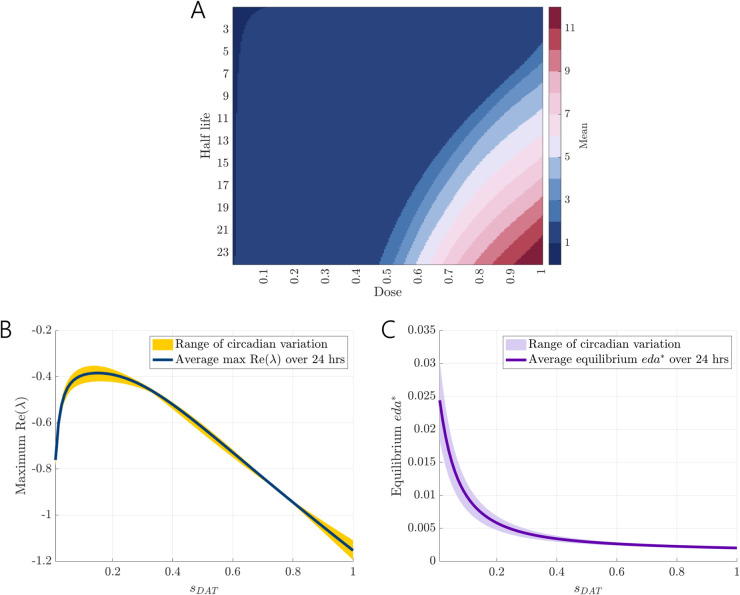
Stable dosing regimes and linear stability analysis. (A) Mean *eda* over 7 days of repeated daily doses at *t* = 6. The change in mean *eda* is robust to a large range of half-lives and doses. The mean *eda* monotonically increases with both half-life and dose, with steep changes outside of the homeostatic plateau (large blue region). (B) Largest real part of the eigenvalues obtained from linear stability analysis over a full 24-hour circadian cycle, evaluated under varying levels of $s_{DAT}\in [0.01,1]$. The yellow shaded region represents the range of maximum real eigenvalues across circadian phases for each value of *s*_*DAT*_. The blue line denotes the average of these eigenvalues at each $V_{DAT}$ activity level. The consistently negative values indicate that the system’s equilibrium remains locally stable across all circadian phases and drug inhibition levels $s_{DAT}\in [0.01,1]$. (C) Average equilibrium eda* across circadian phases for $s_{DAT}\in [0.01,1]$. The thick purple line shows the mean over 24 hrs, and the shaded region indicates circadian variation. Equilibrium eda* decreases monotonically with increasing $V_{DAT}$ activity, reflecting convergence to lower steady states as drug effects decay.

We expected the influence of DRI administration in the model on extracellular dopamine concentrations to depend on the half-life of the administered drug. In Sect 2.2, we assumed a half-life of 15 hours for doses that initially inhibit 0.2 or 0.5 of the dopamine transporters. These doses had time-dependent effects on the temporal dynamics of *eda*, with some dose/dose-time combinations causing large spikes followed by a rapid decline and others allowing *eda* to be sustained for longer. In the differential equations, most of the extracellular dopamine is taken back up into the cell, at a rate of $V_{DAT}$. With longer half-lives, repeated daily doses of DRIs can cause *eda* to accumulate rapidly. We computed mean *eda* over 7 days of repeated doses at *t* = 6 for half-lives between 1 and 24 hours and doses between 0 and 1 (0% and 100% initial inhibition of *DAT*). The average concentration of *eda* did not change more than two-fold for a large range of half-lives and doses; see Fig 5A.

Next, we performed a linear stability analysis of the nominal model described by Eqs (15)–(18), incorporating a scaling factor $s_{DAT}\in [0,1]$ applied to $V_{DAT}$ to simulate the pharmacodynamic effect of DRIs at specific time points. A value of *s*_*DAT*_ = 1 corresponds to the absence of drug effect, while values less than 1 represent increasing degrees of transporter inhibition following drug administration. The time-dependent drug effect was previously modeled using an exponential decay, and here *s*_*DAT*_ reflects the instantaneous $V_{DAT}$ activity at any given time.

To assess how stability depends on dosing phase, we swept the circadian phase ϕ∈[0,24h) that modulates $C_{TH}(\phi )$ and $C_{MAO}(\phi )$. At each pair $\left(s_{DAT},\phi \right)$ we treated the clock parameters as fixed (taking the quasi-static assumption that the clock varies more slowly than dopamine dynamics), solved for the biologically feasible equilibrium $y^{*}(s_{DAT},\phi )=(ldopa^{*},cda^{*},vda^{*},eda^{*})$, and evaluated the Jacobian matrix $J\left(y^{*}(s_{DAT},\phi )\right)$ at equilibrium. Numerical calculations indicate that this feasible equilibrium is unique for every $\left(s_{DAT},\phi \right)$. Details of calculations can be found in the Matlab code in the code repository. We then derived the characteristic polynomial $P(\lambda )=\det\left(J\left(y^{*}(s_{DAT},\phi )\right)-\lambda I\right)$, and solved for its roots to assess local stability.

Fig 5B presents the average of the largest real parts of the eigenvalues across all ϕ∈[0,24h) for *s*_*DAT*_ values ranging from 0.01 to 1. The shaded yellow region indicates the range of these maximum real eigenvalues over the circadian cycle for each level of $V_{DAT}$ activity. At very low values of *s*_*DAT*_, particularly when the value approaches zero, certain circadian phases such as ϕ=13h produce positive real eigenvalues. For instance, when *s*_*DAT*_ = 0 or *s*_*DAT*_ = 0.0001, the system exhibits local instability at this specific phase. However, despite these outliers, the dominant eigenvalue remains negative across most circadian phases, suggesting that the timing of DRI dosing has a limited impact on overall system stability. Importantly, all eigenvalues remain negative across the full range of simulated conditions in the majority of cases, confirming that the equilibrium state (see Fig 5C) is locally stable under varying degrees of $V_{DAT}$ activity and circadian modulation. Thus, although these equilibria represent transient approximations under constant effect of circadian phase, they provide valuable insight into the dynamics and stability characteristics of the system under circadian modulation.

Fig 5C shows the average equilibrium eda*, similarly across all ϕ∈[0,24h) for *s*_*DAT*_ values ranging from 0.01 to 1. The shaded light purple region illustrates the extent of circadian variation. We observe that the average equilibrium eda* decreases monotonically with increasing $V_{DAT}$ activity. Although the time-dependent drug effect is analytically complex, Fig 5C demonstrates that as the drug effect exponentially decays according to the drug’s half-life, *eda* progressively converges towards its equilibrium state, which steadily decreases. This behavior aligns with findings presented in Fig 3, showing that following a single dose, *eda* smoothly converges to its equilibrium.

While our primary linear stability analysis was performed at equilibrium points for fixed *s*_*DAT*_ and *ϕ*, this approach approximates the behavior of the system along actual drug decay trajectories. This is because as the drug effect wanes over time, the system’s state evolves along a sequence of slowly changing equilibria determined by the instantaneous value of *s*_*DAT*_. In this quasi-static regime, evaluating the Jacobian at each of these equilibria effectively captures the system’s local stability along the drug decay trajectory. Therefore, although we do not explicitly compute the Jacobian along a full time-course trajectory (Fig 3A and Fig 4A), our analysis in Fig 5C implicitly reflects this evolution, as it shows how the system tracks the moving equilibrium as *s*_*DAT*_ decays. These findings are consistent with the results shown in Fig 5A, which demonstrate robust system output across a wide spectrum of dosing regimens and drug half-lives.

### 2.4. Generation of ultradian oscillations via coupling to dopaminergic network

We extended our reduced dopamine model by adding two additional Eqs (20) and (21) to explore the potential for intrinsic ultradian dopamine oscillations driven by neuronal population-level feedback. This Dopamine Ultradian Oscillator (DUO) framework is motivated by experimental observations of ultradian rhythms in behavior and physiology [39,51], which are hypothesized to originate from collective dopaminergic activity in neuronal populations.

**Fig 6:**
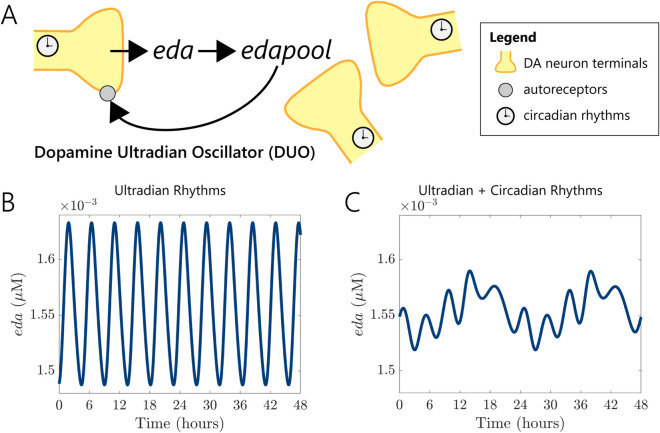
Schematic representation and simulation of Dopamine Ultradian Oscillator (DUO) demonstrating ultradian rhythms with and without circadian modulation. (A) Schematic illustration of the DUO mechanism. *eda* diffuses locally from dopaminergic neuron terminals into a collective dopamine pool (*edapool*). Elevated dopaminergic tone subsequently activates autoreceptor signaling (via *D*2 autoreceptors), leading to negative feedback inhibition of further *eda* release. (B) Simulation depicting pure ultradian rhythms of *eda* levels over 48 hours in the absence of circadian modulation. The rhythmic pattern is consistent and stable. (C) Simulation illustrating ultradian rhythms of *eda* levels within a circadian framework, showing fluctuations where dopamine concentrations are relatively lower during the sleep and relatively higher during wakefulness. Parameters for simulations in (B) and (C) were $k_{1}^{pool}=4519.93$, $k_{2}^{pool}=0.38$, $k_{3}^{pool}=22.3$, $k_{4}^{pool}=2.34$, α=0.5, β=0.5, γ=193.93, *m* = 1.1.

In the DUO model, extracellular dopamine (*eda*) diffuses from multiple dopaminergic neuron terminals into a shared extracellular dopamine pool (*edapool*), representing local dopaminergic tone; see Fig 6A. Elevated *edapool* levels activate autoreceptor-mediated signaling (*D*2), which provides delayed negative feedback on dopamine release. This model structure enables intrinsic ultradian rhythms independent of external circadian input. Using a representative parameter set (see details in Sect 4.5), we observed stable oscillations in *eda* with an ultradian period of approximately 4.6 hours in the absence of circadian modulation (Fig 6B).

We then examined the behavior of the DUO under circadian regulation of *TH* and *MAO* activity as in Sect 2.1. The ultradian rhythm persisted, maintaining a similar period of 4.6 hours, but was now modulated by the slower 24-hour circadian cycle (Fig 6C). Both rhythms were clearly evident: the circadian rhythm shaped the underlying time course of dopamine, with lower *eda* concentrations during simulated sleep intervals and higher levels during wakefulness, while the ultradian oscillations emerged as modulations around the primary circadian signal. Notably, the amplitude of ultradian oscillations varied over the circadian cycle, becoming more pronounced when dopamine synthesis was elevated, illustrating a dynamic interaction between the two rhythmic processes.

In previous sections, we assumed that the 24-hour variation in *eda* was solely governed by circadian regulation of *TH* and *MAO*, and thus did not account for ultradian influences. However, incorporating ultradian rhythms introduces additional complexity, as both the amplitude and period of these rhythms vary considerably across individuals—a variability observed in behavioral and dopaminergic ultradian oscillations, which lack the intrinsic stability of circadian rhythms and are highly responsive to changes in dopamine tone [39,51]. To reduce this complexity and focus on core dynamical features, we opted to use a fixed $V_{DAT}$ activity level, *s*_*DAT*_, rather than modeling a time-dependent drug concentration. This simplification allows us to systematically explore how different steady-state levels of reuptake inhibition affect ultradian dynamics, without the confounding variability introduced by dose timing and individual-specific ultradian profiles. While a time-dependent dosing model may be appropriate in some contexts, it offers limited interpretability here due to the intrinsic variability of ultradian rhythms.

**Fig 7:**
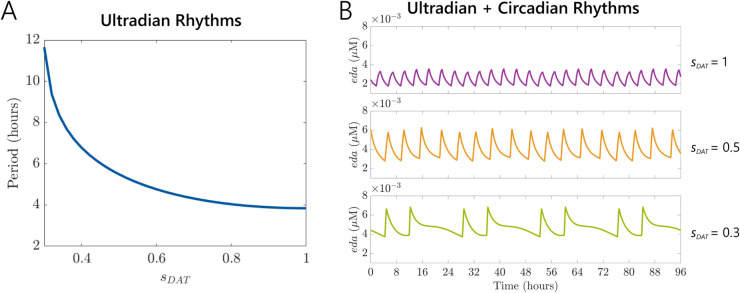
Influence of drug-induced inhibition on the period of ultradian rhythms and their modulation within circadian cycles. (A) Dependence of the ultradian rhythm period on varying drug $V_{DAT}$ activity levels (*s*_*DAT*_) ranging from 0.3 to 1. The period monotonically decreases as the $V_{DAT}$ activity level increases. (B) Simulation of ultradian rhythms modulated by circadian rhythms at selected $V_{DAT}$ activity levels: *s*_*DAT*_ = 1, *s*_*DAT*_ = 0.5, and *s*_*DAT*_ = 0.3. These examples illustrate distinct rhythmic dynamics over a 96-hour timeframe. Parameters used in simulations for (A) and (B) were $k_{1}^{pool}=21118.5$, $k_{2}^{pool}=1.89$, $k_{3}^{pool}=34.45$, $k_{4}^{pool}=0.47$, α=0.5, β=4.5, γ=2139.25, *m* = 0.01.

Building on this approach, we next explored how DRIs influence ultradian periodicity by varying the $V_{DAT}$ activity level (*s*_*DAT*_ from 0.3 to 1) and calculating the resulting ultradian periods (Fig 7A). As reuptake was increasingly inhibited, the ultradian period lengthened from close to 4 hours towards 12 hours, consistent with experimental findings that DRIs slow ultradian behavioral rhythms [39]. We also examined how ultradian-circadian interactions changed under different DRI levels (Fig 7B). At lower inhibition (*s*_*DAT*_ = 1), dopamine oscillations retained a shorter, more regular ultradian rhythm. As *s*_*DAT*_ decreased (i.e., stronger inhibition), the oscillations became broader in period and more irregular in amplitude. These changes reflect a complex interplay between the intrinsic dynamics of the DUO and the entraining influence of the circadian rhythm. Overall, our results suggest that DRIs modulate both the frequency and amplitude of dopamine ultradian rhythms, and that these effects are further shaped by the phase and strength of circadian inputs.

### 2.5. Bifurcation analysis of the Dopamine Ultradian Oscillator (DUO)

In this section, we characterize the limit-cycle dynamics of the DUO model through linear stability analysis in the absence of circadian modulation. Initially, we numerically computed the steady-state solution, denoted as $z^{*}(s_{DAT})=(ldopa^{*},cda^{*},vda^{*},eda^{*},edapool^{*},D2^{*})$, from the system described by Eqs (15)–(21). Subsequently, the Jacobian matrix, $J_{DUO}(z^{*},s_{DAT})$, was analytically derived using Mathematica (details of the derivation can be found in the Mathematica notebook in the code repository). Given that the steady-state z* explicitly depends on the parameter *s*_*DAT*_, we substituted $z^{*}(s_{DAT})$ back into $J_{DUO}(z^{*},s_{DAT})$ for each selected value of *s*_*DAT*_.

We then derived the characteristic polynomial of the Jacobian matrix:


$$P_{DUO}(\lambda_{DUO},s_{DAT})=\det(J_{DUO}(z^{*},s_{DAT})-\lambda_{DUO}I).$$

**Fig 8:**
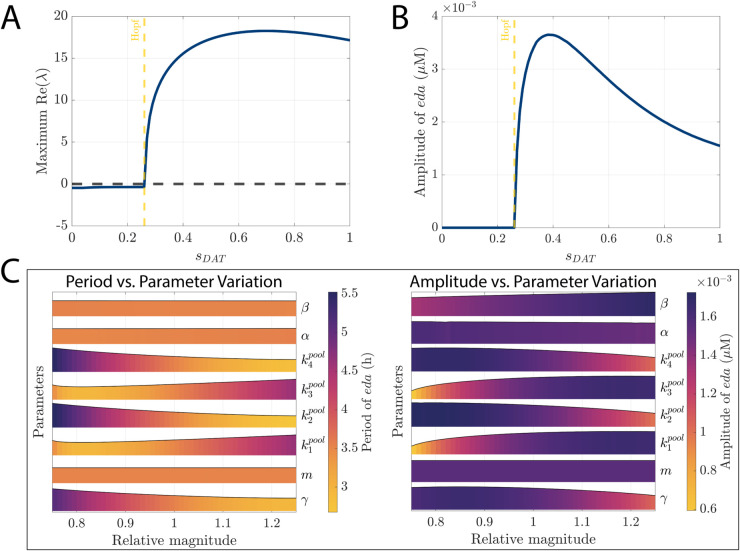
Bifurcation analysis of the Dopamine Ultradian Oscillator (DUO) with respect to *s*_*DAT*_. (A) Maximum real part of eigenvalues of the Jacobian matrix as a function of *s*_*DAT*_ demonstrate the presence of a Hopf bifurcation at *s*_*DAT*_ = 0.26. (B) Non-monotonic relationship between *s*_*DAT*_ and the amplitude of *eda* oscillations. As *s*_*DAT*_ decreases, amplitude initially increases, peaking at *s*_*DAT*_ = 0.42. Further reductions in *s*_*DAT*_ sharply diminish oscillation amplitude, ultimately abolishing ultradian behavior at sufficiently low *DAT* activity. (C) Sensitivity analysis showing stable ultradian rhythms across individual parameter variations (0.75–1.25 × baseline), demonstrating DUO model robustness. The baseline parameters used in simulations were the same as in Fig 7.

Solving for roots numerically, we identified the eigenvalues corresponding to each *s*_*DAT*_. Our analysis shows that the eigenvalues possess nonzero imaginary parts across the entire range of $s_{DAT}\in [0,1]$, indicating oscillatory behavior. Importantly, the largest real part of the eigenvalues transitions from negative to positive as *s*_*DAT*_ decreases through the critical value *s*_*DAT*_ = 0.26, indicating a Hopf bifurcation point (Fig 8A). Biologically, this result implies that large-enough inhibition of DAT activity (lower *s*_*DAT*_ values) can disrupt the ultradian rhythmic behavior inherent to the system.

To further elucidate the impact of dopamine reuptake inhibition on the oscillatory dynamics, we examined the amplitude of *eda* oscillations as a function of *s*_*DAT*_. As illustrated in Fig 8B, the oscillation amplitude exhibits a non-monotonic relationship with *s*_*DAT*_. Specifically, the amplitude increases with decreasing *s*_*DAT*_ until it reaches a maximum at approximately *s*_*DAT*_ = 0.39. Beyond this point, further reduction of *s*_*DAT*_ sharply decreases the amplitude, ultimately abolishing ultradian rhythms altogether. Thus, our model predicts that while moderate inhibition of DAT can increase the amplitude of ultradian fluctuations, excessive inhibition destabilizes and suppresses these rhythmic patterns.

Besides evaluating drug effects, we also examined the sensitivity of the DUO model to variations in all other parameters. To quantify this sensitivity, we introduced a relative magnitude as a scalar multiplier for each parameter in the DUO model. Each parameter was individually varied from 0.75 to 1.25 times its original baseline value. For each adjusted parameter set, we numerically simulated the DUO model over a duration of 100 days. The system’s behavior during the final 4 days of each simulation was recorded to represent the steady-state response.

We generated ridge plots to visualize the amplitude and period of the *eda* oscillations for parameter sets corresponding to Fig 7. Similar plots for parameter sets shown in Fig 6 are provided in S1 Fig. As illustrated in Fig 8C, varying each parameter within the 0.75 to 1.25 range consistently resulted in non-zero amplitudes and periods, confirming the robustness of ultradian rhythmic behavior in the model. Additionally, both amplitude and period did not change dramatically, suggesting that ultradian rhythms in our model can persist despite natural parameter fluctuations.

## 3. Discussion

In this paper, we reduced a detailed model of dopamine synthesis, release, and reuptake [9] from 9 to 4 state variables, demonstrating that the reduced model displays similar, biologically realistic dynamics related to autoregulatory mechanisms. We had previously created mathematical models studying the circadian regulation of dopamine metabolism [23,24,29]. Several studies suggest that drugs targeting the dopaminergic system and dopamine transporters will have time-of-day effects [52,53] and experimentalists have demonstrated time-of-day changes in locomotor sensitization to cocaine [54]. As a result, we used the reduced model to explore time-of-day effects of DRIs, showing that administration in the early morning during circadian troughs allow dopamine levels to be sustained for much longer. Dopamine is important for executive functions [48], and clinical studies have suggested that modafinil is most effective during early morning [49,50].

In general, we did not find many other studies investigating the time-of-day effects of drugs targeting the dopaminergic system. However, our mathematical model provides strong evidence that time-of-day effects in the dopaminergic system could be quite important and underscores the need for further experimental work in this area. In our model, when DRIs are administered during the circadian trough of dopamine synthesis, dopamine concentrations transiently go up since dopamine reuptake is blocked. Then, as the effects of the DRIs wear off towards the natural circadian peak of dopamine synthesis, dopamine levels are sustained. Potential experimental studies could measure DRI efficacy at different time points throughout the day in relation to endogenous rhythms (e.g. melatonin, cortisol, body temperature).

In addition, clinical studies on modafinil timing [49,50] focus on alertness and performance during sleep deprivation, but DRIs other than modafinil can be prescribed for various conditions, including ADHD, depression, and addiction [17]. Thus, “DRI efficacy” can be defined in various ways. Human studies on modafinil and attention might track behavioral measures such as the psychomotor vigilance task (PVT). Brain imaging techniques can be used to determine dopamine activity, such as after sleep deprivation [55]. Different DRIs will have different pharmacokinetic properties, and we find that half-life has a substantial effect in the model. Animal studies have measured extracellular dopamine or dopamine metabolite levels under various experimental conditions targeting the dopaminergic system [56,57]. We believe that the circadian timing of drugs targeting the dopaminergic system is an exciting yet understudied area that can be explored in both human and animal studies. With the availability of new experimental data, future modeling work can explore the effects of drug absorption, DAT binding affinity, and other pharmacokinetic parameters. Based on our model, we hypothesize that treatments near circadian troughs of dopamine are most effective at sustaining dopamine levels.

We found that extracellular dopamine concentrations are remarkably robust to dopamine reuptake inhibitors for a wide range of doses and half-lives (see Fig 5), and once the dose and half-life were large enough, dopamine concentrations increased rapidly. This dynamical feature was expected, as many homeostatic systems display sensitivity outside of some homeostatic range. Our model could be used to study how changes in parameters, such as those related to gene polymorphisms, could influence the homeostatic range and thus the efficacy of DRIs. It’s worth noting that the circadian effects in our model are in relation to the circadian rhythms of dopamine driven by the chosen variation in *TH* and *MAO* activity. While the circadian trough of dopamine may often occur in the early morning, circadian rhythms are highly variable across individuals and even day-to-day within the same individual. Thus, it is important that experimental studies on the timing of DRIs consider measuring circadian markers such as cortisol for reference.

Furthermore, we show that the model can be modified minimally to investigate ultradian rhythms in DA. The DUO is thought to be an important driver of behavioral and physiological ultradian rhythms in mammals [35,36], but the underlying mechanisms are not well understood. We showed that, by coupling our reduced model with an extracellular dopamine pool, the system is capable of robust ultradian rhythms with a period of about 4 to 12 hours. As dopamine reuptake is inhibited, the ultradian period lengthens, aligning with experimental findings [39]. With this extension, our model also provides a novel framework for understanding complex interactions between circadian and ultradian rhythms in the dopaminergic system and how they modulate dynamical changes in dopamine.

## 4. Methods

### 4.1. Model reduction

The model of dopamine synthesis, release, and reuptake by Best et al. [9] consists of 9 ordinary differential equations describing changes in the concentrations of variables: dihydrobiopterin (*bh*_2_), tetrahydrobiopterin (*bh*_4_), tyrosine (*tyr*), l-3,4-dihydroxyphenylalanine (*ldopa*), cytosolic dopamine (*cda*), vesicular dopamine (*vda*), extracellular dopamine (*eda*), homovanillic acid (*hva*), and tyrosine pool (*tyrpool*). The equations are provided below.

$$\frac{d(bh_{2})}{dt}=V_{TH}(tyr,bh_{4},cda,eda)-V_{DRR}(bh_{2},NADPH,bh_{4},NADP)$$ (1)

$$\frac{d(bh_{4})}{dt}=V_{DRR}(bh_{2},NADPH,bh_{4},NADP)-V_{TH}(tyr,bh_{4},cda,eda)$$ (2)

$$\frac{d(tyr)}{dt}=V_{TYRin}(b_{tyr}(t))-V_{TH}(tyr,bh_{4},cda,eda)-k_{1}\cdot tyr+k_{-1}\cdot tyrpool-k_{tyr}^{catab}\cdot tyr$$ (3)

$$\frac{d(ldopa)}{dt}=V_{TH}(tyr,bh_{4},cda,eda)-V_{AADC}(ldopa)$$ (4)

$$\frac{d(cda)}{dt}=V_{AADC}(ldopa)-V_{MAT}(cda,vda)+V_{DAT}(eda)-k_{cda}^{catab}\cdot cda$$ (5)

$$\frac{d(vda)}{dt}=V_{MAT}(cda,vda)-fire(t)\cdot vda$$ (6)

$$\frac{d(eda)}{dt}=fire(t)\cdot vda-V_{DAT}(eda)-V_{CATAB}(eda)-k_{rem}\cdot eda$$ (7)

$$\frac{d(hva)}{dt}=k_{cda}^{catab}\cdot cda+V_{CATAB}(eda)-k_{hva}^{catab}\cdot hva$$ (8)

$$\frac{d(tyrpool)}{dt}=k_{1}\cdot tyr-k_{-1}\cdot tyrpool-k_{tyrpool}^{catab}\cdot tyrpool$$ (9)

The reaction rates are given by

$$V_{TH}=\left(\frac{0.56}{1+\frac{tyr}{K_{i,tyr}}}\right)\left(\frac{4.5}{8{\left(\frac{eda}{0.002024}\right)}^{4}+1}\;+\;0.5\right)\left(\frac{V_{max,tyr}\cdot tyr\cdot bh_{4}}{tyr\cdot bh_{4}+K_{tyr}bh_{4}+K_{tyr}K_{bh_{4}}\left(1+\frac{cda}{K_{i,cda}}\right)}\right),$$ (10)

$$V_{DRR}=\frac{V_{max,bh_{2}}\cdot bh_{2}\cdot NADPH}{\left(K_{m,bh_{2}}+bh_{2})(K_{m,NADPH}+NADPH\right)}-\frac{V_{max,bh_{4}}\cdot bh_{4}\cdot NADP}{\left(K_{m,bh_{4}}+bh_{4})(K_{m,NADP}+NADP\right)},$$ (11)

$$V_{TYRin}=\frac{V_{max,b_{tyr}}\cdot b_{tyr}(t)}{K_{m,b_{tyr}}+b_{tyr}(t)},\;\;V_{AADC}=\frac{V_{max,ldopa}\cdot ldopa}{K_{m,ldopa}+ldopa},$$ (12)

$$V_{MAT}=\frac{V_{max,cda}\cdot cda}{K_{m,cda}+cda}-k_{out}\cdot vda,\;\;V_{DAT}=\frac{V_{max,eda}\cdot eda}{K_{m,eda}+eda},$$ (13)

$$V_{CATAB}=\frac{V_{max,catab}\cdot eda}{K_{m,catab}+eda},$$ (14)

where *NADPH* = 330 and *NADP* = 26 are cofactors for the DRR reaction and all other parameter values are provided in Table 1. The steady state concentrations of the full model are $bh_{2}^{*}=41,\;bh_{4}^{*}=319,\;tyr^{*}=126,\;ldopa^{*}=0.36,\;cda^{*}=2.65,\;vda^{*}=81,\;eda^{*}=0.002,\;hva^{*}=7.69,$ and $tyrpool^{*}=910.61$.

**Table 1:** Parameter values in full model ($\left[K_{◻}\right]$ = *μ*M, $\left[V_{max,◻}\right]$ = *μ*M/hr, and $\left[k_{◻}\right]$ = 1/hr).

Parameter	Value	Parameter	Value
$V_{max,bh_{2}}$	200	*K*_*m*,*NADPH*_	75
$K_{m,bh_{2}}$	100	$V_{max,bh_{4}}$	80
$K_{m,bh_{4}}$	10	*K*_*m*,*NADP*_	75
$V_{max,b_{tyr}}$	400	$K_{m,b_{tyr}}$	64
*k*_1_	6	*k*_−1_	0.6
$k_{tyr}^{catab}$	0.2	$V_{max,ldopa}$	10,000
*K*_*m*,*ldopa*_	130	$V_{max,cda}$	7082
*K*_*m*,*cda*_	3	*k*_*out*_	40
$V_{max,eda}$	8000	*K*_*m*,*eda*_	0.2
$k_{cda}^{catab}$	10	$V_{max,catab}$	30
*K*_*m*,*catab*_	3	*k*_*rem*_	400
$k_{hva}^{catab}$	3.45	$k_{tyrpool}^{catab}$	0.2
*K*_*i*,*cda*_	110	*K*_*i*,*tyr*_	160
*K*_*tyr*_	46	$V_{max,tyr}$	125
$K_{bh_{4}}$	60		

We reduced the model from 9 to 4 state variables by making several simplifications to the biological assumptions. First, we assume the substrate tyrosine is relatively stable in the cell (at steady-state, tyr=126μM in [9]). It is known that tyrosine availability in the blood has a minimal influence on dopamine synthesis, suggesting homeostatic mechanisms that maintain stable intracellular tyrosine concentrations [58]. In the full model, *tyr* enters into the neuron at a constant rate and exchanges linearly with the tyrosine pool. We take $(tyr{)}^{\prime }=(tyrpool{)}^{\prime }=0$, removing Eqs (3) and (9) from the full model and taking the steady state *tyr* = 126. Note that *tyrpool* does not appear in any of the other equations in the full model.

Then, we fix tetrahydrobiopterin (at steady state, $bh_{4}=319\;\mu$M in [9]) and dihydrobiopterin (at steady state, $bh_{2}=41\;\mu$M in [9]). In the full model, the inhibition of *TH* by *cda* competes with the activation of *TH* by *bh*_4_. The cofactor *bh*_4_ is typically tightly regulated and alterations in concentration are associated with neurological diseases like Parkinson’s and neuroinflammation [59]. Taking $(bh_{4}{)}^{\prime }=(bh_{2}{)}^{\prime }=0$ further reduces our system. This reduction closely approximates the original model since Eqs (1) and (2) couple to the rest of the system via $V_{TH}$ (Eq (10)) and the effect of *bh*_4_ on $V_{TH}$ quickly saturates. Finally, since homovanillic acid (*hva*) is downstream of the system, we chose to omit it in this study. Thus, we were able to reduce the system by a total of 5 state variables. The 4 remaining variables in our reduced model of dopamine synthesis are l-3,4-dihydroxyphenylalanine (*ldopa*), cytosolic dopamine (*cda*), vesicular dopamine (*vda*), and extracellular dopamine (*eda*). Schematic diagrams of both the full and reduced mathematical models are provided in Fig 1 for comparison.

### 4.2. Reduced model equations

The simplifications described in Sect 4.1 allowed for a much smaller, more analytically tractable system of equations. The 4 ordinary differential equations in the reduced model are given by Eqs (15)–(18),

$$(ldopa{)}^{\prime }=V_{TH}\left(cda,eda\right)\;-\;V_{AADC}\left(ldopa\right),$$ (15)

$$(cda{)}^{\prime }=V_{AADC}\left(ldopa\right)-V_{MAT}\left(cda,vda\right)+V_{DAT}\left(eda\right)-k_{cda}cda,$$ (16)

$$(vda{)}^{\prime }=V_{MAT}\left(cda,vda\right)-fire\left(t\right)vda,$$ (17)

$$(eda{)}^{\prime }=fire\left(t\right)vda-V_{DAT}\left(eda\right)-V_{CATAB}\left(eda\right)-k_{eda}eda,$$ (18)

with $V_{TH}$ given by Eq (10) with fixed *tyr* = 126 and *bh*_4_ = 319. Thus, the first factor in Eq (10) is effectively a constant. The second factor models the effect of autoreceptors via *eda* feedback, where $\left(\frac{4.5}{8\left(\frac{eda}{eda^{*}}\right){\;\;}^{4}+1}\;+\;0.5\right)=1$ at steady-state ($eda^{*}=0.002024\;\mu$M) and if *eda* goes above or below the steady state the factor will reduce or increase $V_{TH}$, respectively. With *tyr* and *bh*_4_ as constants, the third factor models the inhibition by *cda*. Based on the parameter values, this effect is relatively small.

The remaining reaction rates are kept the same as in the full model [9] and are provided below:


$$V_{AADC}=\frac{V_{max,ldopa}ldopa}{K_{m,ldopa}+ldopa},\;V_{MAT}=\frac{V_{max,cda}cda}{K_{m,cda}+cda}-k_{out}vda$$

$$V_{DAT}=\frac{V_{max,eda}eda}{K_{m,eda}+eda},\;V_{CATAB}=\frac{V_{max,catab}eda}{K_{m,catab}+eda}.$$

All parameter values for the reduced model were kept the same as in [9] and are provided in Table 2. The parameter choices are explained in detail by Best et al. [9], justified by experimental measurements and observations. Thus, we felt it was appropriate to use them as the baseline for our model. Numerical solutions of the reduced model were computed using ode23s in MATLAB. The MATLAB code is provided at https://github.com/rubyshkim/YaoKim_DA.

**Table 2:** Parameter values in reduced model ($\left[K_{◻}\right]$ = *μ*M, $\left[V_{max,◻}\right]$ = *μ*M/hr, and $\left[k_{◻}\right]$ = 1/hr).

Parameter	Value	Parameter	Value
*K*_*i*,*tyr*_	160	$V_{max,ldopa}$	10,000
$V_{max,tyr}$	125	*K*_*m*,*ldopa*_	130
*K*_*tyr*_	46	$V_{max,catab}$	30
$K_{bh_{4}}$	60	*K*_*m*,*catab*_	3
*K*_*i*,*cda*_	110	$V_{max,eda}$	8000
$V_{max,cda}$	7082	*K*_*m*,*eda*_	0.2
*K*_*m*,*cda*_	3	*k*_*cda*_	10
*k*_*out*_	40	*k*_*eda*_	400

In Sect 2.1, we multiply $V_{TH}$ by $C_{TH}(t)=0.25\sin\left(\frac{\pi }{12}(t-12)\right)+1$ and $V_{CATAB}$ by $C_{MAO}(t)=0.25\sin\left(\frac{\pi }{12}(t-20)\right)+1$ to model the circadian variation in enzyme activity, where the phase shifts and amplitudes were chosen to match detailed models of circadian control of dopamine synthesis [23,24].

### 4.3. Reduced model preserves important dynamical behaviors

We investigated numerical solutions to confirm that important dynamical features related to autoregulation were preserved. In the full model [9], equations and parameters were carefully determined based on known kinetics and experimental measurements. A schematic diagram of the model is provided in Fig 1A. In our reduced model (Fig 1B), we used the same functional forms and parameters and expected to get similar steady state concentrations. The steady state concentrations in *μ*M of the variables in the reduced model, ldopa=0.36,cda=2.65,vda=80.96, and *eda* = 0.002, are identical to the concentrations reported in the full model with the exception of *vda* which is 99.9% of the full model’s concentration. These values are also biologically plausible. Experimental measurements suggest that extracellular dopamine in the midbrain is typically in the low nanomolar range [60–62]. At steady state, the model concentration of vesicular dopamine is about 4.6 orders of magnitude larger than *eda*. About 97% of intracellular dopamine (*cda*  +  vda) is stored in the vesicles in the model, consistent with experimental studies [63,64].

**Fig 9:**
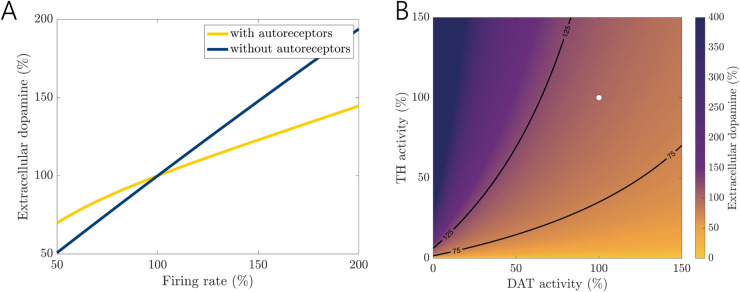
Homeostasis in reduced mathematical model. The reduced mathematical model displays the same homeostatic features as in the full model. (A) The autoreceptors allow extracellular dopamine (*eda*) to be relatively robust to changes in firing rate. The *eda* concentration is plotted as a percentage of the nominal steady state value. (B) *eda* is homeostatic to changes in enzyme activity, that is, the $V_{\max}$ of the reactions catalyzed by *TH* and *DAT*, within the 75–125% homeostatic region (contour lines). Outside this band, particularly at low activity levels, changes in *eda* become much more drastic. The white dot corresponds to the nominal model. Values are plotted as percentages of nominal model values.

The full model constructed by Best et al. [9] displays dopamine homeostasis to firing rate and enzyme activity due to autoreceptor feedback, modeled via the second term in $V_{TH}$, Eq (10). Our reduced model almost exactly reproduces the homeostatic behavior studied in [9] in response to changes in firing rate. In the simulations, firing rate is adjusted by multiplying a function *fire*(*t*) in the equations for *vda* and *eda*. In the nominal model, fire(t)=1. In the absence of autoreceptors in both the full and reduced models, *eda* is sensitive to firing rate, where a 2-fold increase in firing rate causes a 2-fold increase in extracellular dopamine concentration. With autoreceptors, the *eda* curve is much more robust to changes in firing rate; see Fig 9A, which is almost identical to Fig 9 (lower panel) in [9].

In addition, our reduced model displays similar behaviors as in the full model [9] in response to changes in enzyme activity. *TH* is the rate-limiting enzyme in dopamine synthesis and autoreceptors reduce fluctuations in *eda* caused by changes in *TH* activity [9]. In simulations, we modify the maximum rates of the *TH* and *DAT* reactions by 0-150% of their nominal values and calculate the corresponding steady-state value of eda; see Fig 9B compared to Fig 11 in [11].

### 4.4. Dopamine reuptake inhibitors

We modeled the effects of dopamine reuptake inhibitors (DRIs) by adding an additional variable for the concentration, *x*_*dose*_(*t*). The effect of *x*_*dose*_ is that it inhibits the reuptake of dopamine back into the cell. We model this inhibition simplistically by multiplying $V_{DAT}$ by $\max\{1-x_{dose}(t),0\}$ in Eqs (16) and (18), so that when *x*_*dose*_ = 1 there is no reuptake and when *x*_*dose*_ = 0 reuptake happens at the normal rate. In addition, *x*_*dose*_ decays exponentially,

$$x_{dose}^{\prime }=-r\cdot x_{dose}+\sum_{i}Dose\cdot \delta (t-t_{i}),$$ (19)

with *r* chosen appropriately based on measured half-lives of DRIs. In our simulations in Fig 3 and Fig 4A, we chose to model a DRI with a half-life of 15 hours, close to the half-life of the drug modafinil [47], so that $r=\frac{\ln2}{15}=0.0462$. We introduce instantaneous doses of *Dose* at dose times *t* = *t*_*i*_ where δ(t) is the dirac delta function. In simulations, this is equivalent to updating the initial condition for *x*_*dose*_ at the administration times. In the Results, we demonstrate the effects of DRI timing in the model in several different conditions, summarized in Table 3.

**Table 3:** Simulation conditions for the Circadian Time (CT) of DRI administration. In our study, *t* = 0 coincides with CT0.

Simulation condition	Outcome	Dose	Administration times	Half-life
Single dose	Time course of *eda*	0.2 or 0.5	CT6, 12, 18, or 24	15 h
	Mean, median, and SD of *eda*	0.2 or 0.5	CT0 to 24	15 h
Daily doses	Time course of *eda*	0.2 or 0.5	CT6	15 h
	Mean, median, and SD of *eda*	0 to 0.6	CT0 to 24	15 h
	Mean of *eda*	0 to 1	CT6	1 to 24 h

### 4.5. Coupling of model to *eda* pool

We extend our model from the perspective that the DUO is composed of a population of oscillators rather than individual neurons [39,51]. We couple the model to a larger pool of *eda* in the projection region, so that the neuron senses dopaminergic tone. The local *eda* diffuses out, elevating dopaminergic tone, which then promotes autoreceptor signaling (*D*2). The two new ODEs are provided below, where *edapool* and *D*2 are dimensionless, latent variables representing dopaminergic tone and autoreceptor signaling.

$$(edapool{)}^{\prime }=k_{1}^{pool}eda-k_{2}^{pool}edapool$$ (20)

$$(D2{)}^{\prime }=k_{3}^{pool}edapool-k_{4}^{pool}D2$$ (21)

We couple $V_{TH}$ from the original reduced model to the *eda* pool by modifying the second term so that it depends on *D*2. The term $\left(\frac{4.5}{8\left(\frac{eda}{eda^{*}}\right){\;\;}^{4}+1}\;+\;0.5\right)$ in Eq (10) is replaced by

$$𝒜(D2)=\alpha +\frac{\beta }{1+\exp\left(\frac{D2-\gamma \cdot \left(2-s_{DAT}\right)}{m}\right)},$$ (22)

which is a sigmoidal function that decreases with *D*2. In the model, when the *s*_*DAT*_ fraction is reduced from 1, *eda* will build up and *D*2 will also go up, so 𝒜(D2) adapts by shifting to the right. In total, 2 new ODEs and 8 new parameters are added to describe coupling of the dopamine model to dopaminergic tone.

The precise mechanisms of dopamine ultradian oscillations remain elusive [51]. As a result, *edapool* and *D*2 are dimensionless and do not represent precise quantities, but are latent variables accounting for feedback from dopaminergic tone. We chose parameter values using Latin hypercube sampling with several assumptions. First, we took $k_{1}^{pool}>k_{eda}=400$, accounting for contributions of dopamine from nearby dopaminergic terminals. Dopamine concentrations vary widely across brain regions [65,66], so we allowed for changes across three orders of magnitude, $400<k_{1}^{pool}<40,000$. The remaining $k_{i}^{pool}$ for i=2,3,4 were chosen to be small in comparison, within the range [0.1,40], and the parameters *α* and *β* were chosen to keep 𝒜 qualitatively close to the autoreceptor term in the original model (Eq (10)), in the range [0.5,4.5]. The parameter γ∈[100,10000] was chosen to shift 𝒜 to have effects in the solution range of *D*2, and m∈[0.005,5] adjusted the steepness of 𝒜. Within these conditions, parameters leading to stable limit cycle solutions were considered.

## Supporting information

S1 Fig (PDF)
