# Supplementary material for: Mathematical modeling of dopamine rhythms and timing of dopamine reuptake inhibitors
Source: PLoS Comput Biol. 2025 Sep 25;21(9):e1013508. doi: 10.1371/journal.pcbi.1013508 (PMC12494296; doi:10.1371/journal.pcbi.1013508)
Supplement: S1 Fig — (PDF) [file pcbi.1013508.s001.pdf]

Mathematical modeling of dopamine rhythms and timing of dopamine reuptake inhibitors
S1 Fig

Tianyong Yao[1] and Ruby Kim[1,2,*]

[1]Department of Mathematics, University of Michigan, Ann Arbor, Michigan, United States of America

[2]Department of Anesthesiology, Michigan Medicine, Ann Arbor, Michigan, United States of America

[*]rshkim@umich.edu

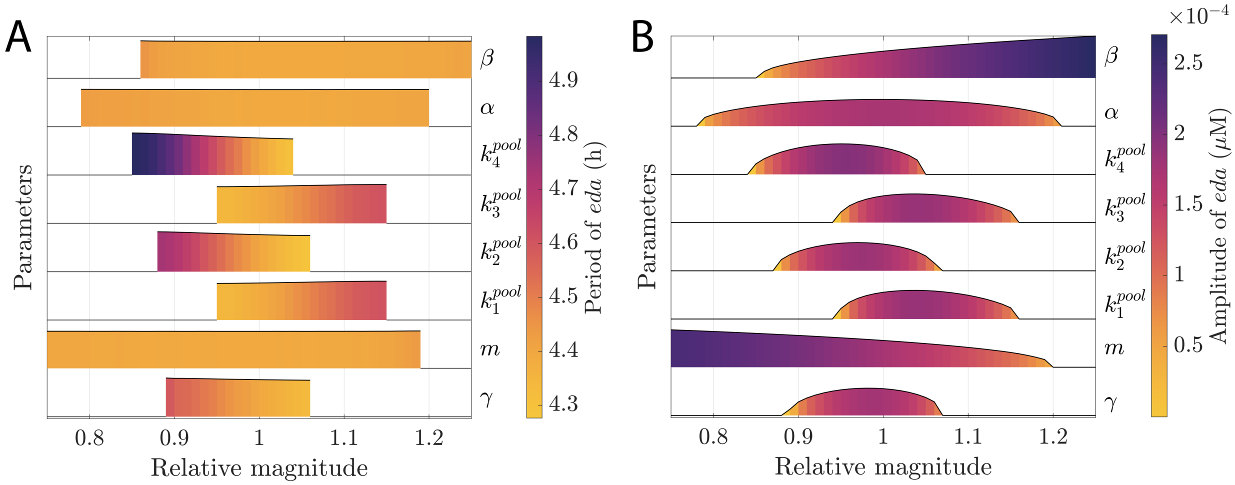

S1 Fig: **Sensitivity analysis of the DUO model for the parameter set used in Fig 6.** Ridge plots illustrate the impact of parameter perturbations (0.75–1.25× baseline) on the period and amplitude of *eda* oscillations. While parameters α, β, and m allow moderate variability without disrupting oscillatory behavior, the model exhibits increased sensitivity to changes in k_i^{pool} $(i = 1, 2, 3, 4)$ and γ. Notably, small perturbations in k_4^{pool} can abolish oscillations entirely. Unlike the robust behavior observed in Figure 7, the limit cycle here is less resilient to parameter fluctuations. The period remains relatively stable across perturbations in α, β, γ, and m, though the amplitude varies significantly. This non-robust parameter regime may represent physiological conditions under which ultradian rhythms are more vulnerable to disruption, potentially modeling pathological dopaminergic states. Physiologically, this sensitivity could help explain the diversity of ultradian rhythm expression observed across individuals.
